# Effect of Channel Interaction on Vocal Cue Perception in Cochlear Implant Users

**DOI:** 10.1177/23312165211030166

**Published:** 2021-08-30

**Authors:** Waldo Nogueira, Nawal El Boghdady, Florian Langner, Etienne Gaudrain, Deniz Başkent

**Affiliations:** 1 Department of Otolaryngology, Medical University Hannover and Cluster of Excellence Hearing4all, Hanover, Germany; 2 Department of Otorhinolaryngology, University Medical Center Groningen, University of Groningen, Groningen, Netherlands; 3 Research School of Behavioral and Cognitive Neurosciences, 3647 University of Groningen, University of Groningen, Groningen, Netherlands; 4 Lyon Neuroscience Research Center, CNRS UMR 5292, INSERM U1028, University Lyon 1, Lyon, France

**Keywords:** channel interaction, cochlear implant, speech-on-speech, F0, VTL

## Abstract

Speech intelligibility in multitalker settings is challenging for most cochlear implant (CI) users. One possibility for this limitation is the suboptimal representation of vocal cues in implant processing, such as the fundamental frequency (F0), and the vocal tract length (VTL). Previous studies suggested that while F0 perception depends on spectrotemporal cues, VTL perception relies largely on spectral cues. To investigate how spectral smearing in CIs affects vocal cue perception in speech-on-speech (SoS) settings, adjacent electrodes were simultaneously stimulated using current steering in 12 Advanced Bionics users to simulate channel interaction. In current steering, two adjacent electrodes are simultaneously stimulated forming a channel of parallel stimulation. Three such stimulation patterns were used: Sequential (one current steering channel), Paired (two channels), and Triplet stimulation (three channels). F0 and VTL just-noticeable differences (JNDs; Task 1), in addition to SoS intelligibility (Task 2) and comprehension (Task 3), were measured for each stimulation strategy. In Tasks 2 and 3, four maskers were used: the same female talker, a male voice obtained by manipulating both F0 and VTL (F0+VTL) of the original female speaker, a voice where only F0 was manipulated, and a voice where only VTL was manipulated. JNDs were measured relative to the original voice for the F0, VTL, and F0+VTL manipulations. When spectral smearing was increased from Sequential to Triplet, a significant deterioration in performance was observed for Tasks 1 and 2, with no differences between Sequential and Paired stimulation. Data from Task 3 were inconclusive. These results imply that CI users may tolerate certain amounts of channel interaction without significant reduction in performance on tasks relying on voice perception. This points to possibilities for using parallel stimulation in CIs for reducing power consumption.

Cochlear implants (CIs) are devices that can restore hearing in patients suffering from profound hearing loss. Although many CI users obtain good speech performance in quiet, their speech intelligibility drops significantly in the presence of a background interfering signal known as the cocktail-party scenario (e.g., Cullington & Zeng, 2008; El Boghdady et al., 2019; Friesen et al., 2001; Fu et al., 1998; Stickney et al., 2004). There are two major masking mechanisms that could contribute to the poor performance of CI users in the presence of background interference. The first is energetic masking, which concerns peripheral auditory processing in the sense that energy components from both the foreground (target) and background (masking) signals overlap spectrotemporally. The second masking mechanism, known as informational masking, is related to more central auditory processes, such as linguistic similarity that might exist between two competing speech signals. Thus, a special category of such cocktail-party scenarios, which includes both types of masking mechanisms, is one in which a target speech signal is masked by a competing speech masker. This setup is considered to be more representative of cocktail-party environments (Assmann & Summerfield, 2004; Bronkhorst, 2000; Brungart, 2001; Duquesnoy, 1983; Festen, 1993; Festen & Plomp, 1990).

Unlike normal-hearing (NH) listeners, who have been shown in the literature to benefit from spectral dips or temporal modulations in a fluctuating masker to obtain release-from-masking (Cullington & Zeng, 2008; Duquesnoy, 1983; Festen & Plomp, 1990; Gustafsson & Arlinger, 1994; Nelson et al., 2003), CI users do not seem to make use of such dips. Evidence for this comes from a number of studies which have demonstrated that CI users have more difficulty understanding speech in the presence of a fluctuating competing speech masker compared to a steady-state noise masker (Cullington & Zeng, 2008; Stickney et al., 2004). In fact, as the number of competing talkers in the masker increases, the spectrotemporal fluctuations in the masker begin to flatten, and hence the multitalker speech masker starts resembling a steady-state masker. In this situation, NH listeners start experiencing more difficulties “*listening in the dips*” of the masker; however, CI users usually find this situation more favorable in comparison to scenarios involving a single-talker masker (Chen et al., 2020; e.g., Cullington & Zeng, 2008).

A possible explanation for these reported perceptual differences between NH and CI listeners may be attributed to the ability of the former group to use voice cue differences that exist between multiple simultaneous talkers in such speech-on-speech (SoS) scenarios (e.g., Brungart, 2001; Cullington & Zeng, 2008; Darwin et al., 2003; El Boghdady et al., 2019; Stickney et al., 2004). On the contrary, CI users do not appear to benefit from such voice differences (Cullington & Zeng, 2008; El Boghdady et al., 2019; Stickney et al., 2004). In particular, the performance of CI users in SoS settings has been shown in a previous study (El Boghdady et al., 2019) to be correlated with their sensitivity to two important voice cues defining the voices of the target and masker speakers: the fundamental frequency (F0) and the VTL of the speaker. The data demonstrated that CI users who were more sensitive to both F0 and VTL cues, and not to only one of them, were more likely to perform better on a number of SoS-related tasks compared to those participants who were sensitive to either cue alone. While F0 and VTL cues are not the only characteristics that define a speaker’s voice (Abercrombie, 1967; Johnson, 2005; Kreiman et al., 2005), this study focuses primarily on these two cues because of their direct link with the anatomy of the human speech production system and because manipulations of these two cues can influence the perceived gender of the speaker (Fuller et al., 2014; Hillenbrand & Clark, 2009; Skuk & Schweinberger, 2014; Smith & Patterson, 2005).

The speaker’s F0 induces the percept of voice pitch and is usually lower for adult males than for adult females (Peterson & Barney, 1952; Smith & Patterson, 2005). These F0 cues are usually encoded in both the temporal envelope (e.g., Moore, 2008) and the cochlear location of excitation (e.g., Carlyon & Shackleton, 1994; Licklider, 1954; Oxenham, 2008), which gives these cues a spectrotemporal nature. The VTL correlates with the speaker’s physical (Fitch & Giedd, 1999) and perceived height (Ives et al., 2005; Smith et al., 2005) and is usually longer for adult males than for adult females. VTL cues are usually encoded in the speech spectral envelope (Chiba & Kajiyama, 1941; Fant, 1960; Lieberman & Blumstein, 1988; Müller, 1848; Stevens & House, 1955). Shortening VTL results in the stretching of the spectral envelope toward higher frequencies on a *linear* frequency scale, while elongating VTL results in the compression of the spectral envelope toward lower frequencies. On a *logarithmic* frequency scale, shortening VTL leads to a translation of the formant peaks in the spectrum toward higher frequencies, while elongating VTL leads to a translation of the formants toward lower frequencies. This effect directly influences the formant frequency space defining vowel boundaries (Peterson & Barney, 1952; Turner et al., 2009). However, the auditory system relies on the relative spacing between formants to identify vowels rather than the absolute values of the individual formants themselves (for a review, see Johnson, 2005). This means that VTL cues can be largely encoded in the relationship between the peaks in the spectral envelope of the signal. Hence, the adequate representation of both F0 and VTL cues would be expected to require sufficient spectrotemporal resolution.

Information transmitted by the CI is usually spectrotemporally degraded (Fu et al., 1998; Fu & Nogaki, 2005; Henry & Turner, 2003; Nelson & Jin, 2004; Winn et al., 2016). Spectrotemporal resolution in the implant depends on a number of factors, such as the amount of channel interaction between adjacent electrodes and the subsequent effective number of spectral channels (for a review, see Başkent et al., 2016). Because of the conductive fluid filling the cochlea, current spreads between neighboring electrodes resulting in channel interaction (e.g., Boëx et al., 2003; De Balthasar et al., 2003; Hanekom & Shannon, 1998; Shannon, 1983; Townshend & White, 1987), and the subsequent reduction in the effective number of spectral channels: CI listeners do not usually have access to more than eight effective spectral channels (Friesen et al., 2001; Qin & Oxenham, 2003). Increased channel interaction does not only smear spectral envelope cues (Wouters et al., 2015) but may also introduce out-of-phase temporal modulations across adjacent channels thereby distorting the temporal envelope cues (Fielden et al., 2015; Vandali et al., 2005). Because F0 transmission relies on both temporal and spectral (place) cues, it can be suspected that channel interaction would affect the perception of such cues in CI listeners. In addition, VTL cues are also expected to be compromised as these cues are largely represented in the formant peak spacing of the spectral envelope of the signal. Using vocoder simulations of CI processing, Gaudrain and Başkent (2015) have demonstrated that as channel interaction increases (simulated as the shallowness of the vocoder filter slopes), the sensitivity to VTL cues deteriorates. Thus, significant channel interaction may not only impair speech and phoneme perception (e.g., Friesen et al., 2001; Fu & Shannon, 2002; Qin & Oxenham, 2003) but also the transmission of F0 and VTL differences required to separate target and masker speakers in SoS scenarios.

Voice cues can be related to more basic psychoacoustic abilities of CI users. For example, the ability to discriminate amplitude modulation rates may be helpful in using F0 cues in speech understanding-related tasks. Chatterjee and Peng (2008) found a nonlinear correlation between CI listeners’ amplitude modulation rate discrimination thresholds and their performance in F0-contour-based speech intonation recognition. However, amplitude modulation rate discrimination in single channels is limited in CI users compared to NH listeners (e.g., Fraser & McKay, 2012). Moreover, in CIs, multiple channel stimulation and subsequent channel interaction may cause modulation interference that may further limit F0 cues (Fraser & McKay, 2012). This effect may be worsened when presenting these multiple channels simultaneously, such as in the Paired (two pairs of simultaneous channels, with one channel consisting of two adjacent electrodes simultaneously stimulated at a time) or Triplet stimulation strategies (three pairs of simultaneous channels).

This study aims to assess the effects of such channel interaction (and resulting spectral resolution) on SoS and voice cue perception in CI listeners by using simultaneous stimulation of multiple channels to induce various degrees of channel interaction. The effect of this channel interaction on the representation of F0 and VTL cues in the speech signal is also investigated using a 3D model to illustrate the increase in channel interaction caused by various stimulation strategies that differ in the number of simultaneously stimulated channels used. Beyond the purpose of evaluating the importance of spectrotemporal cues for F0, VTL, and SoS perception, there is also a potential benefit in using parallel stimulation, as it was originally proposed in the literature as a method of reducing power consumption (e.g., Büchner et al., 2005; Frijns et al., 2009; Langner et al., 2017). One way of achieving this is to decrease the maximum stimulation current required to stimulate the auditory nerve. For instance, by stimulating two adjacent electrodes in the cochlea, it is possible to reduce the amount of current by half to achieve the same loudness percept as that from single electrode stimulation, as the current is distributed between both electrodes. In addition, it is possible to introduce simultaneously stimulated parallel channels, such as Paired and Triplet stimulation, to reduce the maximum current delivered by the implant by a factor of 17% and 44%, respectively. With Paired stimulation, it is possible to double the pulse duration with respect to Sequential stimulation (one pair of simultaneous channels) without changing the stimulation rate of the implant. In terms of performance, Langner et al. (2017) showed no degradation in speech performance under stationary background noise for Paired stimulation compared to Sequential stimulation. However, the same study also showed that increasing the number of parallel channels to three, such as in Triplet stimulation, causes a significant drop in speech intelligibility in comparison to Sequential stimulation. From these results, it was suggested that Paired stimulation may be a good candidate for reducing power consumption in CI users without significant loss in speech-in-noise intelligibility; however, more detailed speech performance measures are required to assess the potential effects of adding parallel channels on CI users’ performance on a number of speech-related tasks. Thus, another goal of this study, if only degradations were to be observed, was to determine the level of parallel channel stimulation that could be acceptable for voice cue and SoS perception, without significant reduction in performance.

Three research questions were addressed in this study: (a) whether increasing the number of parallel stimulated channels (from Sequential to Paired to Triplet stimulation), thereby increasing channel interaction, decreases the sensitivity to F0 and VTL differences in CI users, which was measured using JNDs in Task 1; (b) whether this effect is also reflected as a reduction in SoS perception (Tasks 2 and 3); and (c) whether some parallel channel stimulation could be deployed for reducing power consumption without significantly impairing voice cue and SoS perception. In Tasks 2 and 3, SoS perception was measured as a function of systematically increasing ΔF0 and ΔVTL between target and masker speakers. The setup was designed such that a single-talker target speaker was embedded in a single-talker masker to model one of the more challenging background interference scenarios for CI users as previously mentioned (Chen et al., 2020; Cullington & Zeng, 2008). The target and masker sentences were spoken by the same female speaker to overcome potential confounds related to different speaking styles or rates that may arise from having different speakers (Cullington & Zeng, 2008). The masker’s F0 and VTL values were artificially manipulated to obtain a realistic-sounding male voice. While both Tasks 2 and 3 measured SoS perception, different speech materials were used to measure potentially different aspects of speech perception, namely *intelligibility* and *comprehension*. The participants were also asked to do different activities in each of these tasks as follows. In Task 2, SoS intelligibility was measured in a manner similar to previous literature (El Boghdady et al., 2019, 2020; Pyschny et al., 2011; Stickney et al., 2004, 2007). Participants were asked to repeat all words spoken by the target speaker in the presence of the voice-manipulated masker, and the intelligibility score was determined based on the number of words correctly repeated. In Task 3, a different speech test was administered (sentence verification task [SVT]), which measures overall sentence comprehension (Adank & Janse, 2009; Baddeley et al., 1992; El Boghdady et al., 2019, 2020; May et al., 2001; Pisoni et al., 1987; Saxton et al., 2001). In this task, participants were asked to judge whether the target sentence statement, presented simultaneously with a single competing masker, was true or false, without repeating the actual sentence, and both target sentence comprehension accuracy and speed (response times [RTs]) were measured (Adank & Janse, 2009). An advantage to measuring RTs compared to traditional accuracy (percent-correct) scores is that RTs may help capture subtle differences between experimental conditions that arise from more central auditory processes that may not clearly appear in a typical intelligibility task (e.g., Baer et al., 1993; Gatehouse & Gordon, 1990; Hecker et al., 1966). For example, adverse listening conditions, such as SoS, require a relatively longer time to process and thus lead to longer RTs, compared to ideal listening conditions (Baer et al., 1993; Gatehouse & Gordon, 1990).

The hypotheses were as follows for each research question, respectively: (a) Reductions in sensitivity are expected to increase as a function of increased channel interaction, and should be larger for VTL compared to F0, because VTL is a primarily spectral cue, while F0 cues could still be preserved in the temporal aspect of the signal even if the spectral component is compromised; (b) These reductions in sensitivity for F0 and VTL cues are expected to be reflected as a reduction in both SoS intelligibility and comprehension performance; (c) Some degree of parallel stimulation, such as Paired, may not lead to a significant reduction in voice cue and SoS perception compared to Sequential, which could warrant the use of Paired stimulation as a potential low-power stimulation strategy.

## Methods

### Participants

Twelve native German CI users with Advanced Bionics (AB) devices were recruited from the clinical database of the Medizinische Hochschule Hannover (MHH) based on their clinical speech intelligibility scores in quiet and in noise. To ensure that participants could perform the SoS tasks, the inclusion criteria were to have a speech intelligibility score higher than 70% in quiet and 20% in noise at a +10 dB signal-to-noise ratio on the Hochmair–Schulz–Moser (HSM) sentence test (Hochmair-Desoyer et al., 1997). It is worth noting that, in order to be able to observe effects from voice manipulations on the masker in the SoS tasks, the masker has to be sufficiently audible. For that purpose, from piloting and from previous studies, we have determined that the target-to-masker ratio (TMR) should not exceed +12 dB. In addition, Paired and Triplet stimulation were expected to yield lower performance than Sequential stimulation. With this TMR, and although this may affect the generalizability of our results, we estimated that only better performers would be able to yield performance sufficiently away from floor to have a chance to observe the effects of masker voice and stimulation pattern.

Table 1 shows the demographics of the CI users. All 12 participants took part in both the JND and SoS intelligibility tasks (Tasks 1 and 2), while only 8 (P05–P12) of the 12 participants participated in the SoS comprehension task (Task 3). In the SoS comprehension task, data from P01–P03 were treated as pilot data to better identify the test parameters that would yield reasonable performance away from floor and ceiling effects, and thus could not be included in the final analyses. Retesting these participants with the final test parameters was also not possible because of the anticipation of a learning effect for the SVT materials, and thus all participants were only tested once. P04 found the task difficult and thus opted to discontinue with data collection.

**Table 1.** Demographics for CI Users Recruited.

Participant number	Gender	Age at testing (years)	Implant	Duration of device use (years)	Duration of hearing loss (years)	Etiology	Clinical speech-in-quiet scores
P01	M	20	Helix	4.0	< 1 year	Unknown	100%
P02	F	48	Helix	8.7	0.61	Acute	100%
P03	M	55	Mid-Scala	3.8	Progressive	Unknown	96%
P04	M	58	Mid-Scala	2.5	Progressive	Unknown	100%
P05	M	47	Mid-Scala	5.5	1.5	Acute	100%
P06	M	43	Helix	10.5	Progressive	Acute	98.11%
P07	F	51	Helix	11.4	< 1 year	Genetic	90.56%
P08	F	70	Helix	2.6	5.24	Unknown	100%
P09	M	51	Mid-Scala	5.6	Progressive	Unknown	95.25%
P010	F	46	Helix	9.6	Progressive	Acute	100%
P011	F	49	Helix	8.2	< 1 year	Acute	70.75%
P012	M	65	Helix	10	Progressive	Unknown	99.06%

*Note*. All durations in years are calculated based on the date of testing. Progressive hearing loss refers to participants who experienced minimal hearing loss that gradually progressed until they fulfilled the criteria for acquiring a CI.

### Voice Cue Manipulations

F0 and VTL cues were manipulated relative to those of the original speaker of the corpus in each experiment using the *Speech Transformation and Representation based on Adaptive Interpolation of weiGHTed spectrogram* (STRAIGHT; Kawahara & Irino, 2005). Increasing/decreasing F0 in STRAIGHT is implemented by shifting the pitch contour of the original speech upward/downward by a number of semitones (12th of an octave; st) toward higher/lower frequencies relative to the average F0 of the stimulus. Shortening/elongating VTL is implemented by expanding/compressing the spectral envelope of the signal toward higher/lower frequencies.

**Figure 1.**
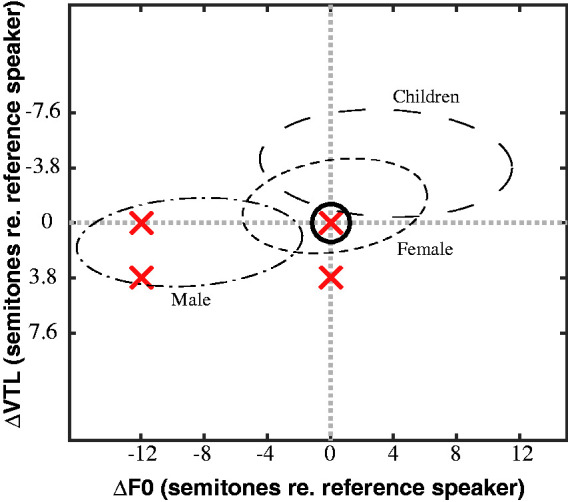
[ΔF0, ΔVTL] Plane, With the Reference Female Speaker From Task 2 Shown as the Solid Black Circle at the Origin of the Plane. Decreasing F0 and elongating VTL yields deeper-sounding male-like voices, while increasing F0 and shortening VTL yields child-like voices. The dashed ellipses are based on the data from Peterson and Barney (1952), which were normalized to the reference female speaker, and indicate the ranges of typical F0 and VTL differences between the reference female speaker and 99% of the population. The red crosses indicate the voice vectors from the origin of the plane along which the JNDs were measured in Task 1, and the four different combinations of ΔF0 and ΔVTL used to construct the maskers in both Tasks 2 and 3. VTL = vocal tract length.

Figure 1 shows the F0 and VTL values (red crosses) used in the current study plotted on the (ΔF0, ΔVTL) plane. The red crosses indicate the voice vectors (directions) from the origin of the plane along which the JNDs were measured in Task 1 (along negative ΔF0, along positive ΔVTL, and along the diagonal passing through ΔF0 = –12 st, and ΔVTL = +3.8 st). In addition, they represent the four combinations of F0 and VTL differences between the masker and target speakers in Tasks 2 and 3. The solid black circle at the origin on the plane indicates the voice of the original female speaker from the corpus used in Task 2. The dashed ellipses encompass the range of relative F0 and VTL differences between the original female speaker and 99% of the population as calculated from the Peterson and Barney (1952) study. This calculation was performed by normalizing the data provided by Peterson and Barney relative to the voice parameters of the original female speaker of the corpus, who had an average F0 of about 218 Hz and an estimated VTL of around 13.97 cm. The original female speaker’s VTL was estimated using the method of Ives et al. (2005) and the data from Fitch and Giedd (1999), assuming an average height of about 166 cm for the speaker based on growth curves for the German population (Bonthuis et al., 2012; Schaffrath Rosario et al., 2011). ΔVTL is oriented upside down to indicate that positive ΔVTLs yield a decrease in the frequency components of the spectral envelope of the signal.

**Figure 2.**
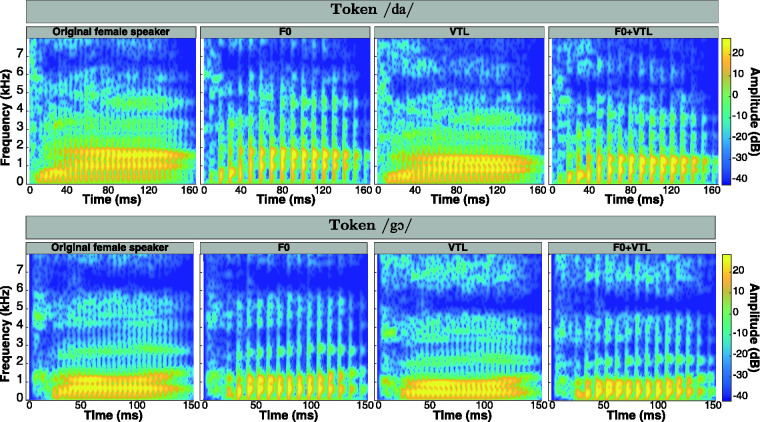
Spectrograms of Two German Tokens [/da/(*top row*) and/gɔ/(*bottom row*)] Shown for Each Voice. *First column from left*: original female speaker from the corpus; *second column from left*: effect of decreasing F0 by 12 st on the spectrogram; *third column from left*: effect of elongating VTL by 3.8 st; *fourth column from left*: effect of both decreasing F0 by 12 st and elongating VTL by 3.8 st relative to the voice of the original female speaker. VTL = vocal tract length.

Figure 2 shows the effect of manipulating F0 and VTL on the spectrograms of two German tokens. The rows represent the different tokens, while the column represent the voice manipulation (no manipulation [original female speaker], F0, VTL, or both F0 and VTL). Notice that as F0 decreases, the number of glottal pulses also decreases, and as VTL is elongated, the spectral content of the signal is compressed toward lower frequencies along a linear frequency scale. In addition, decreasing F0 and elongating VTL together yield less glottal pulses, whose frequency components are also compressed toward lower frequencies.

### F120 Sound Coding Strategies (Sequential, Paired, and Triplet)

#### Fidelity F120 Sound Coding Strategy

**Figure 3.**
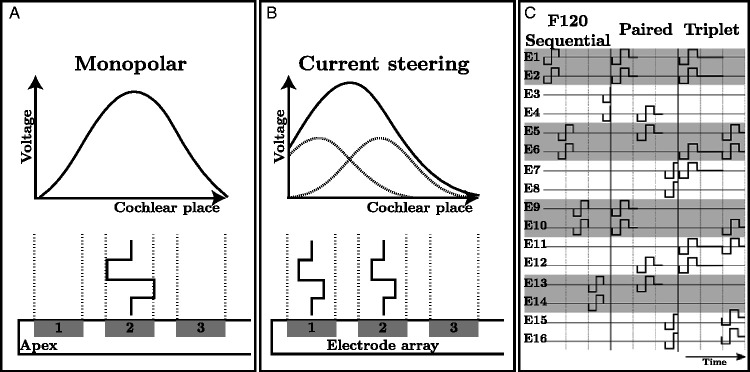
Schematic showing the different stimulation strategies used in the current study.

The Fidelity 120 (F120) in AB devices is a sound coding strategy that processes the audio signal through an automatic gain control. Next, a spectral analysis is performed using a short-time fast Fourier transform to compute the slow varying envelopes in each analysis band. In parallel stimulation, the spectrum is analyzed using a spectral peak locator to estimate the most dominant frequency component in each analysis band. Finally, the slowly varying envelopes are logarithmically compressed into the electric dynamic range of each participant between the threshold and the most comfortable level. Each analysis band is then assigned to two simultaneously stimulated electrodes (Figure 3B). The current ratio between these two electrodes is derived from the spectral peak locator forming a current steered—or virtual—channel. For a given analysis band k, a pair of electrodes are simultaneously stimulated, one with current $I_{k}\cdot \alpha$ and the adjacent one with current $I_{k}\cdot (1-\alpha )$, with $I_{k}$ being the compressed current obtained from the envelope in analysis band k, and α being the current steering coefficient (0≤α≤1) derived from the spectral peak locator. Each analysis band k (k=1…N) is stimulated sequentially (see Sequential stimulation panel in Figure 3C), completing a stimulation cycle. The AB CI has 16 electrodes, and the F120 uses N=15 analysis bands.

Figure 3A provides the concept of monopolar stimulation with its associated voltage spread. Figure 3B demonstrates the concept of current steering (virtual channel) stimulation. With Paired and Triplet stimulation (Figure 3C), each pulse is extended with zero stimulation after the end of the second biphasic pulse to keep the stimulation rate on each channel constant across sound coding strategies.

#### Excitation Patterns Using Sequential, Paired, and Triplet Stimulation

**Figure 4.**
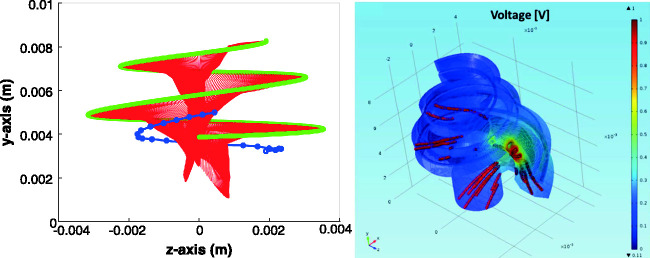
3D finite element model used in the current study.

The effect of spectral smearing using the F120 Sequential, Paired, and Triplet strategies was first analyzed in simulation using a 3D finite element model of the electrically stimulated cochlea (see Nogueira et al., 2016 for details). Figure 4 demonstrates this model as follows.

The spread of electric current in the cochlea is simulated in a 3D finite element method (FEM) from the geometry of the cochlea containing the Scala tympani, Scala vestibuli, Reisner membrane, basilar membrane, the modiolus, and the nerve. The left panel in Figure 4 demonstrates the geometry of the auditory nerve. A spline interpolation of the auditory nerve compartment was used to create 10,000 nerve fibers along the cochlea. The 3D computer-assisted drawing model was generated in Inventor® (Autodesk, San Rafael, CA) and imported into COMSOL Multiphysics® (COMSOL Inc., Burlington, MA) to generate a tetrahedral mesh using the general physics algorithm. An electrode carrier with 16 half-band electrode contacts modeling the HiFocus 1 J was created, as shown by the blue array in the left panel of Figure 4. The physiology of the auditory nerve fiber was modeled as in Ashida and Nogueira (2018). The voltage distribution from the FEM, as shown in the right panel of Figure 4, was sampled at the most peripheral node of the nerve section. For each nerve fiber, the activation function in the most peripheral nerve was computed as in Equation 4 from Nogueira et al. (2016). The current delivered to each electrode across time, also known as electrodograms, for Sequential, Paired, and Triplet stimulation were computed using the F120 sound coding strategy as described in Nogueira et al. (2009). Next, the voltage distribution created by the electrodograms was estimated using the 3D voltage distribution model (see right panel of Figure 4). Finally, the neural excitation patterns, that is, the neural activity across time, were computed using the nerve fiber model described in Nogueira et al. (2016) which is very similar to the one presented by Litvak et al. (2007).

**Figure 5.**
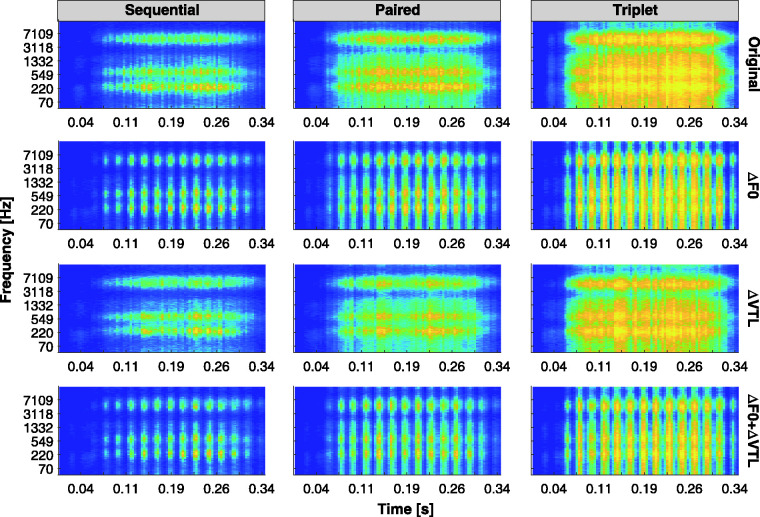
Excitation patterns obtained from the 3D finite element model for the German token /da/.

German tokens were processed with the Sequential, Paired, and Triplet sound coding strategies using the same levels of stimulation (threshold and most comfortable levels). The stimulation patterns served as input to the computational model that estimated the excitation patterns. Figure 5 presents the excitation patterns (number of spikes across fiber number and time) for the German token/da/with the Sequential, Paired, and Triplet strategies. Figure 5 demonstrates that increasing the number of parallel channels causes a clear spectral smearing of the excitation patterns. Changes in F0 are more visible in the temporal dimension, while changes in VTL are more visible in the spectral dimension. Increased channel interaction, as caused by presenting additional simultaneous channels, is hypothesized to have a direct negative effect not only on VTL but also on F0, as comodulation across channels will be increased, potentially smearing the perception of temporal modulation (F0) cues.

For each participant in the study, the F120 Sequential was fitted by adjusting the threshold and most comfortable levels of each electrode individually. Next, the strategy was activated, and the participant was asked to perform a loudness scaling task containing the presentation of a Consultative Committee for International Telephony and Telegraphy (CCITT) noise at 65 dB SPL free field. The threshold and most comfortable levels for the strategy were then adjusted globally until the participant stated a comfortable loudness percept. Afterward, the Paired and Triplet sound coding strategies were fitted by globally adjusting the most comfortable level across all electrodes by the same amount starting from the Sequential map fitting while presenting the same noise signal.

**Figure 6.**
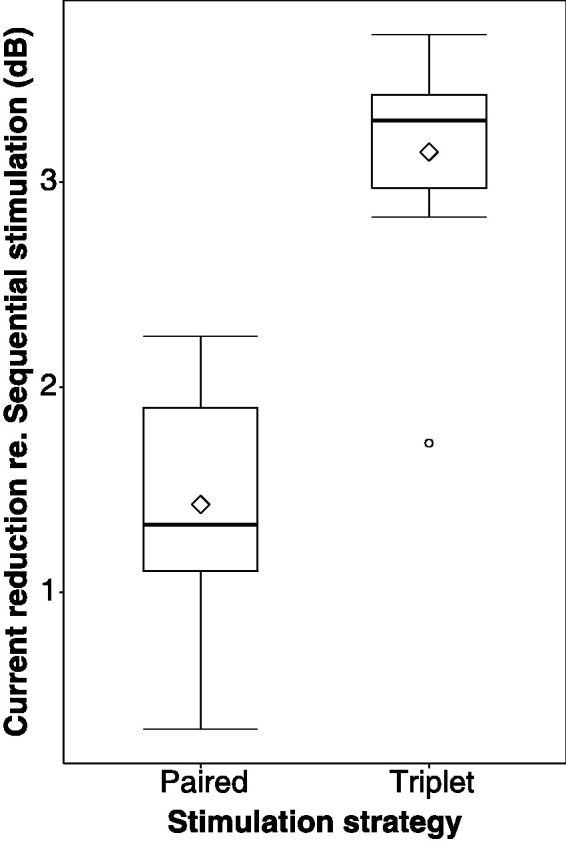
Current reduction in dB when fitting the Paired (left) or Triplet (right) strategies relative to sequential to achieve the same loudness percept.

Figure 6 shows the current reduction in dB for Paired and Triplet relative to Sequential stimulation across all participants recruited in this study. The plot demonstrates that the Sequential strategy requires higher currents compared to either the Paired or Triplet strategies to elicit the same loudness percept and that the Paired strategy requires higher current levels than the Triplet to reach the same most comfortable loudness percept, as was demonstrated by Langner et al. (2017). This is mainly due to the electrical interactions between the simultaneously stimulating channels, decreasing the necessary current required to achieve the same loudness percept (Langner et al., 2020). These interactions depend on the number of stimulating channels and the distance between them. The channel stimulation rate is kept constant across strategies by introducing a nonstimulating zero-phase after the end of the second phase of the biphasic pulse (see Figure 3B). This also implies the possibility for additional power reduction, as an increase in the pulse duration requires much lower current levels to achieve the same loudness percept (Shannon, 1985, 1989) due to the resulting additional spread of excitation (McKay & McDermott, 1999).

In a typical AB device, power consumption is a function of the supply voltage required to power the CI. This supply voltage is determined based on the maximum current the CI can deliver without causing damage to the electrodes. The power consumed by the device is then estimated as the multiplication of the supply voltage with the sum of the leakage current and average stimulation current. The leakage current, reaching around 1 mA in a typical CI, is used to power the implant’s internal circuitry and contributes the largest share of the power consumed. This type of current depends on the technology (transistor sizing) used to manufacture the CI hardware and increases with decreasing transistor sizing. The average stimulation current, however, is bounded by the maximum current level fitted for each CI user. Thus, the most effective method to reduce power consumption is to reduce the supply voltage (Money, 2001; Zeng et al., 2008). Nevertheless, stimulation strategies could also be used to achieve some savings in power consumption. Some previous studies have shown that Paired stimulation reduces average stimulating current by 20% and Triplet by 45% (Langner et al., 2017, 2020).

From this analysis, it can be concluded that adding parallel channels causes current smearing which, in turn, causes a reduction in the current levels required to achieve the same loudness percept, thus achieving the proposed current savings.

### Task 1: F0 and VTL JNDs

#### Stimuli

The methods for this experiment are largely similar to the ones described in El Boghdady et al. (2019) and identical to those in El Boghdady et al. (2020). Therefore, they are described briefly here. Speech material from the Freiburg monosyllabic word test (Hahlbrock, 1953), which consisted of meaningful German monosyllabic words, were rerecorded for this study from an adult native German female speaker. The voice of the speaker had an estimated average F0 of 233 Hz and VTL of 13.9 cm based on her height (164 cm) using the data from Fitch and Giedd (1999). All recordings were equalized in root mean square intensity.

Recordings were made in a sound-isolated anechoic chamber at the University Medical Center Groningen, NL, using a RØDE NT1-A microphone mounted on a RØDE SM6 with a pop-shield (RØDE Microphones LLC, CA, USA). The microphone was connected to a PreSonus TubePre v2 amplifier (PreSonus Audio Electronics, Inc., LA, USA) with noise filtering below 80 Hz. The amplifier output was recorded through the left channel of a DR-100 MKII TASCAM recorder (TEAC Europe GmbH, Wiesbaden, Germany) at a sampling rate of 44.1 kHz. Seventy-five consonant–vowel (CV) syllables were manually extracted from the recorded words in the corpus, resulting in a list of combinations of the consonants (b, d, f, g, h, k, l, l̩, m, n, p, ʁ, z, ʃ, t, v, x, ts) and vowels (iː, oː, uː, a, ɛ, ɪ, ʊ, ɔ, eː).

A single trial consisted of concatenating three random CV syllables, with a 50-ms silence in between, to form a triplet of syllables. Within the trial, the same triplet of syllables was presented three times, with a 250 ms silence gap between each presentation. One of these three presentations was processed to have a different voice (lower F0, longer VTL, or both), as indicated by the vectors from the origin of the (ΔF0, ΔVTL) plane to the red crosses shown in Figure 1. All three presentations were resynthesized with STRAIGHT (Kawahara & Irino, 2005), even when F0 and VTL were not manipulated. The task was to select the triplet that had a different voice with respect to the other two in an adaptive three-interval, three-alternative forced choice task (3I-3AFC).

#### Procedure

Following the paradigm used in a number of previous studies (El Boghdady et al., 2018, 2019, 2020; Gaudrain & Başkent, 2015, 2018), JNDs in this experiment were measured along three voice vectors, as indicated by the red crosses in Figure 1, using a two-down one-up adaptive procedure. This adaptive procedure results in 70.7% correct responses on the psychometric function (Levitt, 1971). A JND measurement consisted of a number of trials: A trial started with the target (voice-manipulated) triplet having a difference of 12 st relative to the other two reference triplets. After the participant’s response, a new trial began with a triplet composed of different combinations of syllables than the previous trial. If the participant was able to correctly detect the voice-manipulated triplet on two consecutive trials, the voice difference between the reference triplets and the voice-manipulated triplet was reduced by 4 st. Otherwise, if the participant was unable to correctly identify the voice-manipulated triplet, the difference between the reference triplets and the voice-manipulated triplet was increased by the same step size. If the difference between the voice-manipulated and reference triplets became less than twice the step size, the step size was reduced by a factor of $\sqrt{2}$. The procedure terminated after eight reversals, and the JND was calculated as the mean of the last six reversals.

The JND measurement for each of the three voice vectors was repeated three times per strategy, resulting in a total of 27 experimental conditions (3 voice vectors × 3 repetitions each × 3 coding strategies). Experimental conditions were blocked per strategy, meaning that a participant would perform all conditions for a given strategy before switching to the next one, and the order of the strategies was randomized per participant. Participants were blinded to the strategies tested.

Training was administered before the beginning of each strategy block with two voice vectors different than those used for data collection: (ΔF0 = +5 st, ΔVTL = –7 st) and (ΔF0 = –12 st, ΔVTL = +3.8 st). Each training condition was terminated after six trials, whether the algorithm had converged or not. Visual feedback was always provided.

### Task 2: Speech-on-Speech Intelligibility

#### Stimuli

Stimuli taken from the German HSM sentence test (Hochmair-Desoyer et al., 1997) were used for the SoS intelligibility task, which is composed of 30 lists with 20 sentences taken from everyday speech, including questions. Sentences in this corpus are made up of three to eight words, with a single list containing 106 words in total. Lists 1–19 were used in this experiment and were previously recorded at the MHH from an adult native German female speaker, who had an average F0 of 218 Hz. All recordings were equalized in root mean square intensity.

Four different masking voices were created as shown in Figure 1: the same talker as the target female speaker (resynthesized with ΔF0 = 0 st, ΔVTL = 0 st), a talker with a lower F0 relative to the target female speaker (ΔF0 = –12 st, ΔVTL = 0 st), a talker with a longer VTL relative to the target female speaker (ΔF0 = 0 st, ΔVTL = +3.8 st), and a talker with both a lower F0 and a longer VTL relative to the target female speaker to obtain a male-like voice (ΔF0 = –12 st, ΔVTL = –3.8 st). These conditions are referred to as *Same Talker*, *F0*, *VTL*, and *F0+VTL*, respectively, in the rest of this article. The parameters for F0 and VTL were chosen based on the findings of an earlier study, in which CI users showed reduced SoS intelligibility and comprehension when the voice of the masker was manipulated with parameters taken from the top-right quadrant in Figure 1 (El Boghdady et al., 2019). In that study, the authors reasoned that masking voices taken from the lower-left quadrant, as performed in the current study, should be expected to yield a benefit in SoS performance for CI users. This was shown to be the case in another later study by the same authors (El Boghdady et al., 2020).

Test sentences were taken from Lists 1–8 and 16–19, while maskers were constructed from Lists 9 and 10. Training sentences were obtained from Lists 11, 12, and 13, with one list randomly assigned per strategy. All sentences assigned for constructing the maskers were processed offline before data collection using STRAIGHT, with all combinations of ΔF0 and ΔVTL highlighted earlier. For the Same Talker condition, the masker sentences were also processed with STRAIGHT, without changing F0 or VTL. All target sentences were kept as the natural, unprocessed version (not processed with STRAIGHT).

Within a trial, the masker sequence started 500 ms before the onset of the target sentence and ended 250 ms after the offset of the target. For the specific ΔF0 and ΔVTL combination within the trial, the masker was constructed from random 1-s-long segments selected from the masker sentences previously processed with STRAIGHT. A raised cosine ramp of 2 ms was applied to the beginning and end of each segment before concatenating them to form the masker sequence. Finally, both the beginning and end of the entire masker sequence were ramped using a 50-ms raised cosine ramp.

Target sentences were calibrated at 65 dB SPL, and the intensity of the masker sequence was adjusted relative to that of the target to obtain the required TMR. The TMRs used for training and data collection in this task were set to +8 dB and +12 dB, respectively, following the protocol of El Boghdady et al. (2019). In that study, the authors demonstrated that a TMR of +8 dB had the potential of capturing group performance in the middle of the psychometric function (away from floor and ceiling effects). The stimuli for all three experiments were sampled at 44.1 kHz, processed, and presented using MATLAB R2014b (The MathWorks, Natick, MA).

#### Procedure

The SoS paradigm for this experiment was based on that used by El Boghdady et al. (2019, 2020). A given trial consisted of presenting a single target-masker combination, and the participant was asked to repeat what they heard from the target sentence. As in Task 1, experimental conditions were blocked per strategy, and the order of the strategies was randomized per participant.

A short training was provided for each strategy block, with both auditory and visual feedback. During the training phase of a given strategy, 12 sentences were randomly selected from the assigned training list: Six sentences were presented in quiet, while the remaining six were presented with a competing masker. The masker voice used for training was assigned different values for ΔF0 and ΔVTL than those used during data collection (–6 st and +6 st, respectively).

Data collection was composed of a total of 240 trials for all three strategy blocks (20 sentences per list × 4 voice conditions × 3 strategies) generated offline prior to the beginning of the experiment. The trials within a strategy block were randomized. No feedback was provided, and the stimulus was presented once. The participants’ responses were scored on a word-by-word basis using a graphical user interface programmed in MATLAB. In addition, the verbal responses were recorded and stored as data files for later offline inspection.

Response words were scored in the following fashion: The German HSM sentences include words that are hyphenated in the corpus, such as “Wochen-ende” (weekend). These words, although written without the hyphen, are hyphenated in the HSM corpus to be scored separately. Only the part repeated by the participant was marked as correct. In addition, the response word was also considered correct if a participant changed the order of the words in the sentence.

A response word was considered incorrect if only a part of the word was repeated for words that are not hyphenated in the HSM corpus, such as saying “füllt” (fills) when the word was “überfüllt” (crowded). In addition, confusion of adjective form, for example, saying “keiner” (“not any” as used with a masculine noun) instead of “keine” (“not any” as used with a feminine noun), or confusing the Dativ with the Akkusativ article, for example, confusing “der” with “dem” or “den,” was also considered incorrect. Confusion of verb tenses or incorrect verb conjugation was considered incorrect. A total of four scheduled breaks were programmed into the experiment script; however, participants were encouraged to ask for additional breaks whenever they felt necessary. The whole procedure lasted for about 1–1.5 hr, including breaks.

### Task 3: Speech-on-Speech Comprehension

#### Stimuli

The voice conditions for the masker in this experiment were the same as those defined in Experiment 1. The masker sequence was created as described in Experiment 1 from Lists 9 and 10 from the HSM material. Target sentences were based on German translations of the Dutch SVT developed by Adank and Janse (2009) and designed to measure sentence comprehension accuracy and speed (RTs). This corpus is composed of 100 pairs of sentences, with each pair composed of a true (e.g., *Bevers bouwen dammen in de rivier* [Beavers build dams in the river]) and false version (e.g., *Bevers grooien in een moestuin* [Beavers grow in a vegetable patch]). All sentences are grammatically and syntactically correct.

Translation from Dutch to German and the evaluation was performed thoroughly by three independent native German speakers: Two of those speakers were also fluent in Dutch, while the third had sufficient knowledge of the language (see El Boghdady et al., 2020, for a full description of the translation procedure). One sentence pair lost its meaning when translated to German and was discarded from the translations, resulting in 99 true–false sentence pairs. The additional four sentence pairs introduced by El Boghdady et al. (2019) for training purposes were translated to German as well.

Recordings were made in the same manner and using the same setup as those described in Task 1. Recordings were taken from an adult native German female speaker, with an average F0 of 180 Hz, and an estimated VTL of about 14.1 cm following the method provided by Ives et al. (2005) and the data from Fitch and Giedd (1999).

#### Procedure

Following the paradigm in previous studies for the SVT (Adank & Janse, 2009; El Boghdady et al., 2020; Pals et al., 2016), participants were asked if the target sentence was true (labeled “WAHR”) or false (labeled “UNWAHR”) by pressing the corresponding button on a button-box as quickly and accurately as possible within a time window of 6 s as soon as they knew the answer. The window was larger than the one used in Pals et al. (2016), to accommodate the CI users and not prime them to guess on most trials. If the time window was exceeded, the response was recorded as a no-response, and the next stimulus was presented. RTs were measured relative to the offset of the resolving word in the stimulus as was done by El Boghdady et al. (2020).

As was done in Tasks 1 and 2, trials were also blocked per strategy, with the order of the strategies randomized across participants. A short training was provided at the beginning of each strategy block. Twelve fixed sentence pairs were assigned for training and were excluded from data collection. Out of these 12 pairs (24 true–false sentences), 4 true and 4 false sentences were randomly picked and assigned to the training block of each strategy. No true–false pair was assigned to the same training block.

In each training block, two true and two false sentences were first presented without a competing masker, followed by the remaining two true and two false sentences, which were presented with a competing masker. This masker also had the same voice parameters as those of the training masker voice used in Task 2 and at the same training TMR of +12 dB. Both audio and visual feedback was provided: Participants were shown if the sentence was true or false, and the sentence was shown on the screen while the whole stimulus was replayed through the loudspeaker.

The remaining sentences (84 true and 84 false sentences) not used for training were used for data collection. These sentences were distributed among the number of conditions tested (4 masker voice conditions × 3 strategies), and no true–false pair was assigned to the same condition. This resulted in seven true and seven false sentences per voice condition per strategy. A test TMR of +10 dB was used in this task because pilot measurements with P01-P03 revealed that a test TMR of +10 dB for this task was expected to yield performance in the middle of the psychometric function compared to a test TMR of +8 dB as was used in Task 2. All stimuli were generated offline for all three strategy blocks and pseudorandomized within each block. During data collection, no feedback was given.

## Results

All data were analyzed using R (Version 3.3.3, R Foundation for Statistical Computing, Vienna, Austria; R Core Team, 2017), and regression models were implemented using the *lme4* package (Version 1.1-15; Bates et al., 2015). When multiple comparisons were carried out, as in the case of the post hoc analyses, a false-discovery rate (FDR) correction (Benjamini & Hochberg, 1995) was then applied to all *p* values obtained from the multiple comparisons.

### Task 1: Effect of Channel Interaction on F0 and VTL JNDs

**Figure 7.**
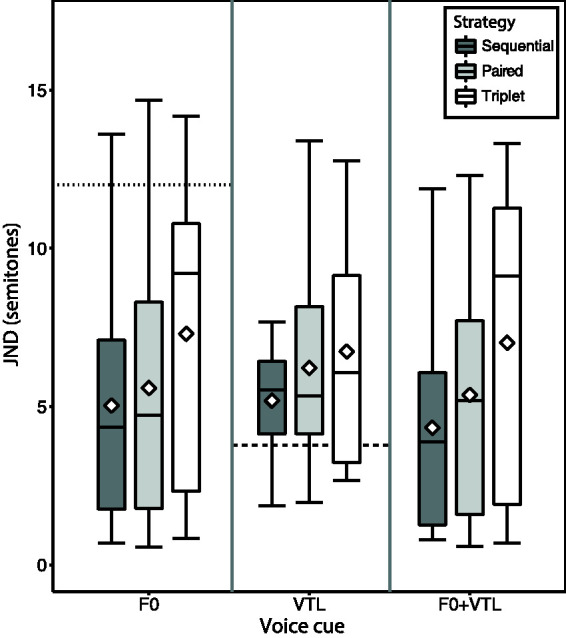
JND Distributions for F0, VTL, and F0+VTL Cues Obtained Under Each Stimulation Strategy: Sequential (Dark Gray Boxes), Paired (Light Gray Boxes), and Triplet (White Boxes). *F0:* JNDs obtained along the negative F0 axis (lowering F0). *VTL:* JNDs obtained along the positive VTL axis (elongating VTL). *F0+VTL:* JNDs obtained along the diagonal with the combination F0 = –12 st, VTL = +3.8 st, simulating a male voice. The boxplot statistics are as indicated in Figure 6. The horizontal dotted line indicates an F0 difference of 12 st as used in the masker setup of Tasks 2 and 3.The horizontal dashed line indicates a VTL difference of 3.8 st as used in the masker setup of Tasks 2 and 3. The asterisk symbol indicates comparisons that yielded significant differences. VTL = vocal tract length; JND = just-noticeable difference.

Figure 7 shows the JND distributions across all participants obtained for each voice cue and indicates, as expected, a trend of worsening (increasing) JNDs as the amount of channel interaction increases (going from Sequential stimulation to Paired to Triplet). To investigate the general effect of channel interaction (stimulation strategy) on voice cue JNDs, a linear mixed-effects model (LMM) was fitted to the log-transformed JNDs. This transformation was performed because the raw JNDs are bounded by zero and thus do not follow a normal distribution. The model was defined with strategy and voice cue (F0, VTL, and F0+VTL), along with their interaction, as the fixed-effect predictors. Interaction effects were included in the model to test whether the effect of strategy changes for different voice cues. Differences in baseline performance between participants, in addition to variations in the effect of strategy from one participant to the other, were accounted for in the linear model as random effects. To quantify the general effect of strategy on JNDs, a one-way type III repeated-measures analysis of variance (ANOVA) was applied to the aforementioned linear model and revealed a significant general effect of strategy on JNDs, *F*(2, 11.21) = 4.70, *p *=* *.03, but no significant differences in JNDs between the different voice cues, *F*(2, 13.35) = 1.98, *p *=* *.18. The interaction effect between strategy and voice cue was also found to be nonsignificant, *F*(4, 55) = 0.91, *p *=* *.47.

A similar LMM (including only a random intercept per participant as the random effect) was applied to each type of JND separately (F0, VTL, or F0+VTL) to study how stimulation strategy (channel interaction) affects each individual voice cue. A similar ANOVA to the one applied on the previous general model was also applied here for each model separately, and *p* values were then adjusted for multiple comparisons using the FDR method (Benjamini & Hochberg, 1995). These ANOVAs revealed that the general effect of strategy observed in the general model arose from a significant effect of strategy on F0 JNDs, *F*(2, 22) = 4.59, *p *=* *.03, and F0+VTL JNDs, *F*(2, 22) = 4.56, *p *=* *.03, but not on VTL JNDs, *F*(2, 22) = 1.23, *p *=* *.31.

The post hoc analyses of these tests revealed that F0 JNDs increased by about 1.44 st as the strategy changed from Sequential to Triplet—β = 0.36, *SE* = 0.13, *t*(22) = 2.87, *p *=* *.03—but did not seem to be affected by Paired stimulation—β = 0.07, *SE* = 0.13, *t*(22) = 0.59, *p *=* *.56. On the contrary, VTL JNDs were neither affected by Paired—β = 0.12, *SE* = 0.12, *t*(22) = 1.01, *p *=* *.39—nor by Triplet stimulation—β = 0.19, *SE* = 0.12, *t*(22) = 1.54, *p *=* *.21—compared to Sequential. Finally, the participants’ JNDs to differences along both F0 and VTL (F0+VTL condition) also significantly increased (worsened) by about 1.35 st when the stimulation strategy was changed from Sequential to Triplet—β = 0.37, *SE* = 0.12, *t*(22) = 3.02, *p *=* *.03—but not from Sequential to Paired—β = 0.19, *SE* = 0.12, *t*(22) = 1.57, *p *=* *.21.

### Task 2: Effect of Channel Interaction on SoS Intelligibility

**Figure 8.**
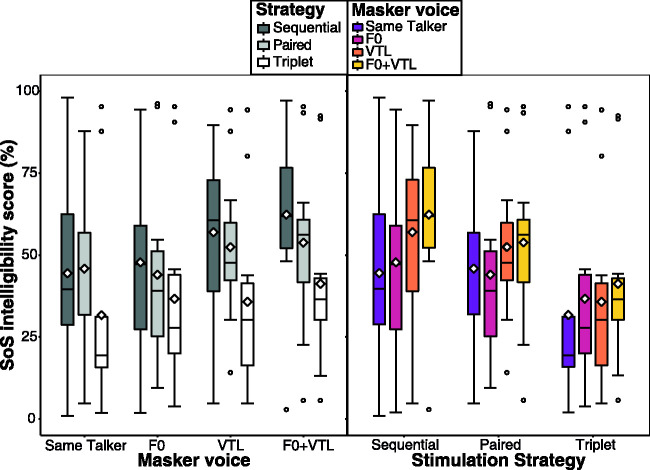
SoS intelligibility scores from Task 2.

Figure 8 shows the distribution of SoS intelligibility scores across participants for each masker voice condition under each stimulation strategy. The scores in this figure were computed as the percentage of correctly repeated words out of the total number of words presented per condition. The average score across all conditions was as low as 46.08%, confirming that it was safer to favor better performers in the experiment design. The data demonstrate that even though there is a large variability in performance across the CI participants for each stimulation strategy (left panel), there appears to be a trend for decreasing SoS intelligibility scores as the amount of channel interaction increases (going from Sequential stimulation to Paired to Triplet). In addition, the representation of the data in the right panel reveals that the degree of benefit in SoS intelligibility scores obtained from changing the masker voice relative to that of the target seems to decrease as the amount of channel interaction increases.

The binary per-word scores (0: incorrect; 1: correct) were modeled using logistic regression as implemented by a generalized linear mixed-effects model (GLMM) with a logit link function. The logistic regression model was fitted to the binary per-word score with strategy and masker voice, along with their interaction, as the fixed-effects. The interaction between stimulation strategy and masker voice was included to test for the significance of the effect observed in the right panel of Figure 8, in which the degree of benefit in SoS intelligibility scores obtained from changing the masker voice seems to diminish as the amount of channel interaction increases. The GLMM was also defined to estimate a random intercept per participant to account for differences in baseline performance across participants. In addition, random effects for strategy per participant and masker voice per participant were also included in the model to account for variations in the effect of strategy and masker voice on SoS intelligibility across participants.

As with the analyses of the JND task, an ANOVA (car package; Fox & Weisberg, 2011) was applied to the GLMM to test for the global effect of strategy, masker voice, and their interaction on the SoS intelligibility scores. Because this ANOVA is applied to a logistic regression model, the output is a table of chi-squared (χ^2^) tests performed on the fixed-effects of the model instead of the traditional *F*-test statistics. The ANOVA revealed a significant effect of stimulation strategy, χ^2^(2) = 27.29, *p *<* *.0001; masker voice, χ^2^(3) = 36.32, *p *<* *.0001; and their interaction, χ^2^(6) = 37.34, *p *<* *.0001.

A post hoc analysis was conducted using an ANOVA applied to the logistic regression model for the effect of strategy under each voice cue separately (left panel in Figure 8) with FDR correction applied to the *p* values. This analysis revealed that SoS intelligibility decreased as a function of increasing channel interaction for the Same Talker condition, χ^2^(2) = 9.34, *p *=* *.01; F0 condition, χ^2^(2) = 8.99, *p *=* *.01; VTL condition, χ^2^(2) = 26.39, *p *<* *.0001; and F0+VTL condition, χ^2^(2) = 34.69, *p *<* *.0001 (see left panel of Figure 8). These effects seemed to arise from the significant reduction in SoS intelligibility under Triplet stimulation compared to Sequential for most voice conditions—Same Talker: β = –0.68, *SE* = 0.23, *z* = –2.92, *p *=* *.009; F0: β = –0.53, *SE* = 0.28, *z* = –1.91, *p *=* *.11; VTL: β = –1.00, *SE* = 0.26, *z* = –3.81, *p *<* *.001; F0+VTL: β = –1.03, *SE* = 0.20, *z* = –5.16, *p *<* *.0001—but not between Paired and Sequential stimulation as obtained from the coefficients of the logistic regression model—Same Talker: β = 0.05, *SE* = 0.22, *z *=* *0.23, *p *=* *.82; F0: β = –0.10, *SE* = 0.22, *z* = –0.44, *p *=* *.75; VTL: β = –0.12, *SE* = 0.26, *z* = –0.45, *p *=* *.75; F0+VTL: β = –0.38, *SE* = 0.24, *z* = –1.59, *p *=* *.18. Consistent with the observations made from the JND task, a reduction in SoS intelligibility was observed with increasing channel interaction for all voice conditions. Thus, as channel interaction increases, spectral features that are important for both voice cue perception and SoS intelligibility appear to be degraded.

The significant interaction effect from the global ANOVA indicates that the benefit in SoS intelligibility obtained from changing the masker voice cues relative to those of the target was affected by the amount of channel interaction: As the channel interaction increased (going from Sequential stimulation to Paired to Triplet), the benefit obtained from the voice differences between masker and target speakers (going from Same Talker to F0 to VTL and then to F0+VTL) decreased significantly (see right panel of Figure 8). To test for the specific benefit from voice differences under each stimulation strategy separately, a similar post hoc analysis was conducted also with FDR correction applied to the *p* values. This post hoc analysis demonstrated a significant benefit in SoS intelligibility from voice differences only for Sequential, χ^2^(3) = 58.27, *p *<* *.0001; and Triplet stimulation, χ^2^(3) = 26.99, *p *<* *.0001, but not for Paired stimulation, χ^2^(3) = 7.08, *p *=* *.07. Under Sequential stimulation, participants were found to gain a significant improvement in SoS intelligibility under the VTL, β = 0.66, *SE* = 0.24, *z *=* *2.76, *p *=* *.02, and F0+VTL conditions, β = 0.97, *SE* = 0.15, *z *=* *6.47, *p *<* *.0001, compared to the Same Talker condition; however, there was no difference in SoS intelligibility between the Same Talker and F0 conditions, β = 0.17, *SE* = 0.18, *z *=* *0.93, *p *=* *.39. The voice benefit observed under the Triplet strategy arose from the significant improvement in SoS intelligibility for the F0+VTL condition compared to the Same Talker condition, β = 0.57, *SE* = 0.13, *z *=* *4.36, *p *<* *.0001, but there was no significant difference between SoS intelligibility for either the F0 or VTL conditions compared to the Same Talker conditions (*p *>* *.08). There was no voice benefit observed under the Paired strategy for F0, VTL, or F0+VTL compared to the Same Talker condition (*p *>* *.08). Together with the data representation in the right panel of Figure 8, this analysis reveals that the degree of benefit in SoS intelligibility scores obtained from changing the masker voice relative to that of the target seems to decrease as the amount of channel interaction increases.

### Task 3: Effect of Channel Interaction on SoS Comprehension Accuracy and RTs

**Figure 9.**
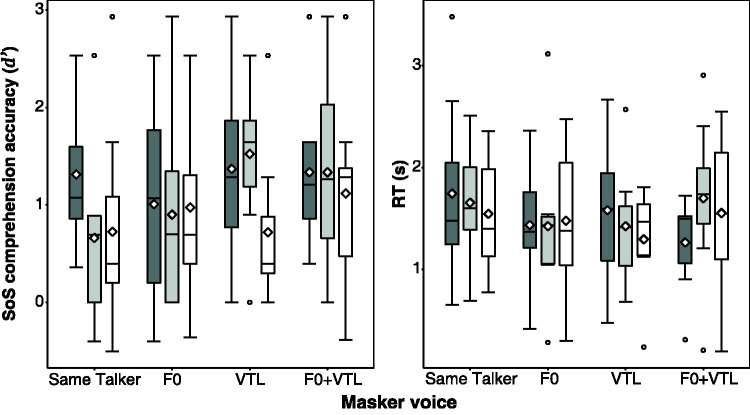
SoS Comprehension Accuracy in *d’* (Left Panel) and RT (Right Panel) for Each Masker Voice Condition Under Each Stimulation Strategy. Boxplot statistics and the description of conditions are the same as those described in the caption of Figure 6. VTL = vocal tract length; SoS = speech-on-speech; RT = response time.

Figure 9 shows the SoS comprehension performance for each masker voice under each stimulation strategy. The left panel shows the effect of strategy on SoS comprehension accuracy converted to the sensitivity measure *d’*, computed as the ratio between the hit rate and the false alarm rate (Green & Swets, 1966). The *d’* measure was used instead of percent-correct because the *d’* is unbiased to a participant’s particular preference for one response at the expense of the other. The large interparticipant variability appears to dilute the effect of strategy. As with the analyses applied to the data of the previous two tasks, an LMM was fitted to the *d’* data with strategy, masker voice, and their interaction as the fixed effects, and a random intercept per participant. Adding random slopes for the effect of strategy per participant and masker voice per participant did not improve the model fit to the data, χ^2^(20) = 15.58, *p *=* *.74, and was thus not included in the final LMM. An ANOVA similar to that applied to the LMM in the JND task was also applied to the LMM modeling the *d’* data and revealed no effect of strategy, *F*(2, 77) = 2.68, *p *=* *.07; masker voice, *F*(3, 77) = 1.82, *p *=* *.15; or their interaction, *F*(6, 77) = 1.20, *p *=* *.31; on the *d’* accuracy scores.

The right panel of Figure 9 shows the RT distributions obtained for each masker voice condition under each of the three stimulation strategies. Again, because of the large interparticipant variability, the effect of strategy on RTs is not evident. Because the RTs considered were those corresponding to only the correct responses, the number of RT data points differed across participants and conditions, which rendered the use of an ANOVA inappropriate. In addition, the RT distributions per participant per condition were largely positively skewed. For these reasons, a GLMM with an inverse Gaussian distribution and inverse link function was fitted to the RT data, as was suggested by Lo and Andrews (2015), and as was carried out by El Boghdady et al. (2019, 2020). The GLMM best fitting the RT data included strategy, masker voice, and their interaction as the fixed-effects, in addition to random intercepts per participant. Including a random slope for strategy and masker voice per participant did not improve the overall model fit (Akaike information criterion [AIC] = 4213.03 and Bayesian information criterion [BIC] = 4362.18 for the model with random slopes versus AIC = 4205.78 and BIC = 4267.19 for the model without random slopes). An ANOVA applied to the GLMM best fitting the RT data did not reveal an effect of strategy, χ^2^(2) = 0.006, *p *=* *.997; masker voice, χ^2^(3) = 0.049, *p *=* *.997; or their interaction, χ^2^(6) = 0.167, *p *=* *.9999, on RTs.

In this task, no effect of strategy could be observed either for SoS comprehension accuracy or RTs. Qualitatively, this implies that participants may be compromising accuracy for speed or vice versa and that these response strategies differ per condition. Consider, for example, the *d’* accuracy scores and RT data for the VTL condition. It appears that as participants give less accurate scores as the channel interaction increases, they also give these responses faster. However, this response strategy seems to change for the condition F0+VTL. In that condition, as channel interaction increases, participants also give less accurate responses, but they do so at slower speeds. Because the analyses yielded nonsignificant effects, no further conclusions could be drawn from this task.

## Discussion

This study investigated whether increasing channel interaction as a result of simultaneously stimulating multiple channels in the CI would lead to a reduced sensitivity to F0 and VTL cues (Task 1) and, correspondingly, reduced SoS intelligibility and comprehension performance (Tasks 2 and 3).

### Task 1: Effect of Channel Interaction on F0 and VTL JNDs

The data from the JND task revealed that, in line with what was expected, increasing channel interaction significantly reduced CI users’ sensitivity to voice cues (both spectral and temporal features), as demonstrated by the main effect of stimulation strategy in addition to a lack of interaction effect between voice cue and stimulation strategy. When mild channel interaction exists, as was the case when Paired stimulation was compared to Sequential, sensitivity to voice cue differences was not significantly affected. However, as the channel interaction increased, as was the case with Triplet stimulation, sensitivity to voice cue differences was reduced. Because no significant interaction effect between stimulation strategy and voice cue was observed in the overall model, the effect of strategy should not be expected to differ for each voice cue. The fact that post hoc analyses revealed no significant effect of strategy on VTL JNDs may have arose from the relatively smaller differences in VTL JNDs across all three strategies compared to the F0 differences, even though a trend for worsening VTL JNDs could be observed. In an earlier study with vocoders, Gaudrain and Başkent (2015) have shown that when the number of effective spectral channels was sufficient, increasing channel interaction (shallower vocoder filters) did not lead to a significant worsening of VTL JNDs. Thus, a possible explanation for these findings could be that the participants tested in the current study already had sufficient effective spectral channels which might have mitigated the detrimental effects of increased channel interaction.

A second observation concerns the effect of channel interaction on F0 JNDs. Because F0 information is encoded in both spectral and temporal cues (Carlyon & Shackleton, 1994), it was expected that the representation of F0 should have been robust to spectral degradations introduced by increased channel interaction. However, F0 cues were shown to be impaired by increased channel interaction, indicating that the temporal aspect of these cues could not provide adequate F0 information for the CI listeners to reach the same JNDs as in the condition of minimal channel interaction (Sequential stimulation). More concretely, and relating the limited perception of F0 cues to more basic psychoacoustic abilities in CI users, it is possible that the increased channel interaction degraded the temporal acuity in CI users caused by interferences on the amplitude modulations conveyed across multiple simultaneously stimulated channels. Another possible explanation for the presence of an effect of channel interaction on F0 JNDs but not for VTL JNDs could be related to the natural differences between male and female voices. Consider the dotted and dashed horizontal lines in Figure 7 for the F0 and VTL JNDs, respectively, which represent the difference between a typical male and female voice for F0 and VTL. Notice that for the F0 JNDs, most participants’ thresholds fall below that typical male–female F0 difference indicating that most of them are sensitive to this voice difference. However, for the VTL cue, most participants have JNDs that are above the typical male–female VTL difference. It may be that VTL cues are already sufficiently degraded even in the Sequential stimulation case such that any added degradation from Triplet stimulation may not yield a difference in results, although a general trend of worsening JNDs could be observed. This may be akin to a floor effect on a speech intelligibility task. Taken together, these findings indicate that an adequate spectral resolution in the implant would be crucial for transmitting both F0 and VTL-related cues.

### Task 2: Effect of Channel Interaction on SoS Intelligibility

The data from the SoS intelligibility task demonstrated an effect of channel interaction, a benefit from voice differences between target and masker speakers, and a significant interaction effect between these two factors. Compared to Sequential stimulation, increasing the channel interaction was shown to impair SoS intelligibility scores only for Triplet stimulation but not for Paired stimulation. This indicates that for mild cases of channel interaction, baseline SoS intelligibility could still be maintained. However, for more extreme cases of channel interaction, as in the case of Triplet stimulation, SoS intelligibility scores become severely degraded.

The significance of the voice benefit under Triplet stimulation is counterintuitive, as it was expected that the severity of the channel interaction would impair the benefit from voice differences compared to less severe cases of channel interaction (e.g., Paired stimulation). This is because the hypothesis is that channel interaction is expected to impair the transmission of voice cues. However, in the Triplet case, SoS intelligibility not only becomes capped but is also severely reduced compared to Sequential (see right panel of Figure 8). In addition, the largest benefit obtained from the condition F0+VTL under Triplet stimulation is almost the same as the mean intelligibility score for the Same Talker condition under either Sequential or Paired stimulation.

These findings reveal that substantial channel interaction may sufficiently degrade the signal to the extent that a benefit in SoS intelligibility from voice cue differences between two concurrent speakers may be impaired. Moreover, consistent with what has been observed in the JND task, CI participants appear to withstand mild channel interaction without a significant drop in their performance levels. However, as the channel interaction becomes more substantial, as is the case when Triplet stimulation is applied, overall SoS intelligibility scores start decreasing dramatically.

Another observation is that the effect of channel interaction on SoS intelligibility as a function of voice differences between the target and masker speakers is the opposite of what can be expected based on the JND data. At a first glance, these findings may seem contradictory to those observed in the JND task because one may expect that as long as participants have useable F0 JNDs, they should be able to gain a larger benefit in SoS from F0 differences compared to VTL differences. This, however, is not the case in the current SoS task, as VTL differences appear to contribute to a larger benefit in SoS situations. A possible explanation for this can be drawn from evidence related to CI users’ performance in speech-on-speech versus speech-in-noise settings. Multiple studies have demonstrated that contrary to NH listeners, CI users can understand target speech better when masked by a noise masker compared to a speech masker (Cullington & Zeng, 2008; Nelson et al., 2003; Stickney et al., 2004). The JND data in the current study provide evidence that the F0 cue is more readily useable compared to the VTL cue. This means that maskers having a different VTL than the target may be less intelligible than those whose F0s have been manipulated with respect to the target. In other words, maskers in the VTL condition may be perceived less like speech maskers and more like noise makers, while maskers in the F0 condition may still be perceived as speech. This indicates that VTL maskers may contribute less to informational masking while F0 maskers could still contribute both to informational and energetic masking, making the VTL maskers less effective and thus yielding the larger SoS intelligibility benefit observed. Another supporting argument for this reasoning is that elongating the VTL of the talker, as was performed in this study, leads to a compression of the spectrum toward lower frequencies which in turn provides less energetic masking of the higher frequency components of the target speech. In an earlier study by El Boghdady et al. (2019), shortening VTL to elicit a child-like voice, contributed to an additional masking effect as CI users’ SoS intelligibility scores dropped as the masker’s VTL was shortened.

The voice parameters for F0 and VTL assigned for the maskers in this study (starting from a female voice and approaching a male-like voice) yielded a benefit in SoS intelligibility. In a previous study (El Boghdady et al., 2019), the authors demonstrated that voice parameters taken from the top-right quadrant of the (ΔF0, ΔVTL) plane (toward child-like voices) failed to provide release from masking for CI users, even though the differences between those child-like voices and the reference female speaker were larger than those between the male-like voices and the reference female speaker in the current study. In another later study (El Boghdady et al., 2020), elongating the masker’s VTL relative to the target speaker to create a male-like voice yielded a benefit in SoS intelligibility for CI users.

Taken together, these data indicate that CI users may benefit differently from voice cue differences depending on which speaker space they cover. However, this benefit from voice differences between target and masker is reduced as the amount of channel interaction increases, as was demonstrated by the significance of the interaction effect observed between stimulation strategy and voice cue. This means that as channel interaction becomes substantial, CI listeners may not be able to benefit from voice cue differences between competing talkers in SoS scenarios.

It is important to mention that CI users may use different acoustic cues that contribute to phonetic perception than those used by NH listeners to obtain word recognition. Winn et al. (2012) suggested that under spectrally degraded conditions, NH listeners decrease their use of formant cues and increase their use of durational cues. Based on these results, they further suggested that although some phonetic cues are obscured by spectral degradation, CI listeners should be able to use nonspectral cues in speech, which might be carried by the temporal amplitude envelope or segment duration. For instance, Winn et al. (2012) showed that CI listeners tend to make less use of the F1 transition and consonant voicing cues and made more use of the vowel duration cue. The current study shows that speech understanding performance with sound coding strategies that introduce simultaneously stimulated channels or sequential stimulation was similar. More concretely, the performance between Sequential and Paired was similar; however, it is possible that CI users changed their listening strategy when listening to the Sequential or Paired stimulation, similar to the strategy changes observed in NH listeners when listening to spectrally degraded sounds by a vocoder (Winn et al., 2012). However, Triplet stimulation showed significantly worse speech understanding than Sequential indicating that even if the phonetic cues extracted were changed, this was not sufficient to compensate the negative effects of increased channel interaction.

It should also be noted that the results presented in this study should be treated as the best-case-scenario when comparing performance of CI users under Sequential, Paired, and Triplet stimulation. This is because higher performing CI users had to be recruited for this study to avoid potential floor effects, as was explained in the Methods section. A larger, more representative sample of CI users should be tested in a follow-up study to better assess the effects of simultaneous stimulation on CI users performing at all levels so that the findings would be more generalizable to a wider range of CI users.

### Task 3: Effect of Channel Interaction on SoS Comprehension Accuracy and RTs

The data from the SoS comprehension were nonconclusive regarding the effect of channel interaction: Comprehension accuracy and RT measures revealed no effect of either channel interaction or voice cue. The observations from this task seem to highlight the different response strategies between listeners. In addition, a large amount of intersubject variability was also observed within this small sample of CI participants, which also limits the scope of conclusions that can be drawn from this task. In order to obtain performance results that can be more generalizable to the larger CI population, a larger sample containing of CI participants should also be tested in upcoming follow-up studies.

## General Discussion

The findings from this study highlight the importance of spectrotemporal resolution when performing tasks that depend on voice-cue perception. This raises the question of whether CIs could be fitted with the goal of mitigating the effect of decreased spectrotemporal resolution that may arise from channel interaction. Several studies (e.g., Di Nardo et al., 2011; El Boghdady et al., 2018; Fitzgerald et al., 2013; Fu & Shannon, 1999; Grasmeder et al., 2014; Leigh et al., 2004; McKay & Henshall, 2002; Omran et al., 2011) have proposed that optimizing the frequency-to-electrode allocation map could have the potential to address the limited spectral resolution in the implant. More specifically, using vocoder simulations, El Boghdady et al. (2018) have shown that the frequency-to-electrode allocation map could have a direct influence on VTL JNDs and that the frequency mapping, if optimally fitted, could help reduce the detrimental effects of channel interaction and frequency mismatch in the cochlea on VTL JNDs. These studies help to pave the way for investigating whether the CI parameters (such as the frequency allocation map) or signal processing could be optimized in a way to improve both SoS perception and the sensitivity to voice cues.

The second goal of this study was to determine the amount of parallel channel stimulation that would not contribute to a significant reduction in performance. While Paired stimulation yielded performance results that were not significantly different from Sequential stimulation on all three tasks, it still remains an open question as to whether Paired stimulation may impact other aspects of sound perception, such as sound quality. For example, Nelson et al. (2018) showed that many hearing aid users adjusted their gain settings to different values than those assigned by an audiologist, probably due to a perceived improvement in comfort and signal quality. However, speech understanding with the two settings was not observed to be significantly different. Thus, additional metrics that more systematically measure listening effort and overall speech quality should be tested before recommending using parallel stimulation as a method for saving battery life in CI devices.

## Conclusion

This study showed that increasing channel interaction by increasing the number of simultaneously stimulated channels significantly reduced CI users’ sensitivity to voice cues (both spectral and temporal features). Compared to Sequential stimulation, increasing the channel interaction was shown to impair SoS intelligibility scores only for Triplet stimulation but not for Paired stimulation. SoS comprehension accuracy and RT measures revealed no effect of either channel interaction or voice cue, although some evidence for a change in participant response strategy could be observed. The lack of a detrimental effect of Paired stimulation on voice cue sensitivity and SoS intelligibility provides evidence that parallel stimulation could be used as a method for reducing power in CIs without impairing performance on tasks relying on voice cue perception.
